# Spread of Phlebotominae in temperate climates: province of Córdoba, Argentina

**DOI:** 10.1590/0074-02760150381

**Published:** 2016-01

**Authors:** Andrés Mario Visintin, Mauricio Daniel Beranek, Maria José Amieva, Juan Ramón Rosa, Walter Ricardo Almirón, Oscar Daniel Salomón

**Affiliations:** 1 Universidad Nacional de Córdoba, Facultad de Ciencias Exactas, Físicas y Naturales, Instituto de Investigaciones Biológicas y Tecnológicas, Centro de Investigaciones Entomológicas de Córdoba, Córdoba, Argentina; 2 Universidad Nacional de La Rioja, Centro de Investigación e Innovación Tecnológica, La Rioja, Argentina; 3 Universidad Nacional del Nordeste, Instituto de Medicina Regional, Resistencia, Argentina; 4 Consejo Nacional de Investigaciones Científicas y Técnicas, Resistencia, Argentina; 5 Ministerio de Salud de Córdoba, Área de Epidemiología, Córdoba, Argentina; 6 Instituto Nacional de Medicina Tropical, Jujuy y Neuquén, Puerto Iguazu, Misiones, Argentina

**Keywords:** Migonemyia migonei, Evandromyia cortelezzii, American cutaneous leishmaniasis, Córdoba

## Abstract

We report the presence of the competent vector for *Leishmania*spp, *Migonemyia migonei*, and the*Evandromyia cortelezzii-sallesi* complex south of its known distribution in the central temperate region of Argentina, in the province of Córdoba. The persistence of this phlebotomine in the northern border of the province, its association with a case of cutaneous leishmaniasis, and the new record in the outskirts of the city of Córdoba, the second most populated in the country, strengthens the need for regular vector surveillance and a case detection-sensitive health system in vulnerable regions, even in temperate climates.

The province of Córdoba is located in the geographical centre of Argentina. The area of the province sampled in this report has a climate gradient from subtropical with a dry season in the northeast corner to temperate in the centre-west up to the foothills, with a mean temperature of 16-17ºC and 750-800 mm of annual precipitation (Jarsún et al. 2003). This bio-region belongs to the Chaco domain and includes the dry Chaco and Espinal sub-domains (Cabrera 1976) (Figure).

**Figure:**
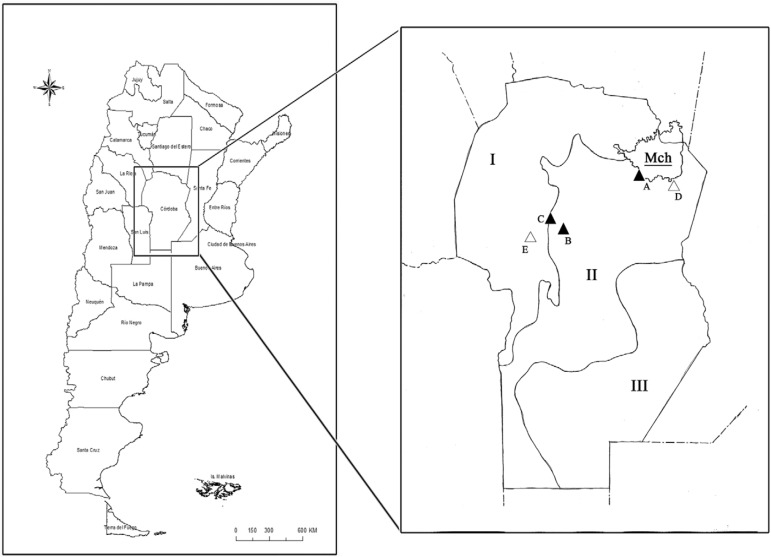
Sampling locations in the province of Córdoba, Argentina (left square: Argentina; right square: province of Córdoba). A: La Para; B: city of Córdoba; C: Unquillo; D: Altos de Chipión; E: Las Jarillas; I: dry Chaco; II: Espinal; III: Pampa; MCh: Mar Chiquita Lagoon; open triangles: historical capture sites 2004-2007 (already published); solid triangles: new captures sites 2012-2015.

The northern border of the province is close to the southernmost known reports of vectorial transmission of American cutaneous leishmaniasis (ACL) in the central region of Argentina, in the province of Santiago del Estero. Despite the fact that Córdoba was monitored for culicid-transmitted arbovirus for more than 40 years, the first reported captures of phlebotomine sandflies in the province were in Altos de Chipión, in 2004/2005, 236 km from the southern record in Santiago del Estero (Los Telares 28º 58’47”S 63º27’04”W), and in Las Jarillas, within the dry Chaco region (Figure, Table) (Salomón et al. 2008). Furthermore, although the Health System regularly reports imported cases of ACL properly diagnosed in Rawson Hospital in the city of Córdoba, the first case suspected to be autochthonous was reported to the National Surveillance System in the 50th week of 2014 (MSAL 2015).

**TABLE:** Phlebotominae reported in the province of Córdoba by date, locality, and species

Locality	Coordinates	Masl	Month/year	Mm M/F	Ecs M/F
Altos de Chipión	30º54’19”S 62º18’11”W	156	Nov/2004-May/2005	11/12	-
Las Jarillas	31º32’02”S 64º33’01”W	758	Nov/2007	-/1	-
La Para	30º51’52”S 62º56’49’’W	89	Apr/2012	-	-/1
	30º51’28’’S 62º55’52’’W		Jan/2012	-	2/-
	30º51’02’’S 62º55’08’’W		Feb/2012	-	2/2
			Apr/2012	-	-/2
			May/2012	-	1/-
			Jan/2012	-	3/-
			Feb/2012	-	5/3
			Apr/2012	-	-/1
			May/2012	-	1/1
City of Córdoba	31º23’38’’S 64º04’36″W	350	Apr/2014	-	-/5
			Feb/2015	-	-/1
Unquillo	31º14’22’’S 64º18’02″W	575	Dez/2014	-/1	-

Ecs: *Evandromyia cortelezzii-sallesi*; M/F: males/females (Ecs males identified as *Ev. cortelezzii*, females of cortelezzii complex); masl: meters above sea level; Mm:*Migonemyia migonei*.

This paper presents three new records of phlebotomine sandflies in the province of Córdoba, their persistence in the northeast humid region and in the Espinal region captured close to Córdoba without previous records, the second most populated city in Argentina (Census 2010: 1,330,023 inhabitants), and captures related to the ACL case.

The captures were made in (i) La Para, 64 km from former captures at Altos de Chipión and sharing the basin of Mar Chiquita Lagoon (salt water). CDC miniature light traps baited with CO_2_ were placed in four sites (3 with sandflies) between December 2011-May 2012, every 15 days. The sites are humid areas with vegetated or rural landscapes with cows and wild foxes in the surroundings; (ii) the city of Córdoba, where the traps were located in three sites: the Zoo (31º25’34’’S 64º10’33’’W), the Botanical Garden (31º23’13’’S 64º14’58’’W), and the “Bajo Grande” sewage treatment plant (31º23’38’’S 64º04’36’’W), the last being the only one with phlebotomines. “Bajo Grande” is located in the outskirts of the city 10 km from downtown in a nonurbanised area, where four CDC traps baited with CO_2_ and separated from each other by 500 m were working seasonally for two nights from 2013-2015. The environment is close to the Suquía River border and has trees and grass, a house 100 m from the traps with roaming dogs and chickens, and wild animals such as foxes, hares, rats, and other rodents; (iii) the city of Unquillo, with six traps located in henhouses, pigsties, and horse stables. The single trap with sandflies was from the henhouse in the yard of the ACL case. The former two trappings were made during arbovirus surveillance study designs, the last one for the focus study of the ACL case. Further captures were made in the course of surveillance for the spread of leishmaniasis vectors in urban and periurban environments during February 2013 (CDC light trap, 2 consecutive nights) at 10 sites at Altos de Chipión and seven sites at Dean Funes (30º26’S 64º21’W), but no phlebotomines were captured. Species were identified with a microscope using Galati’s key (Galati 2003).*Evandromyia cortelezzii* and *Evandromyia sallesi*females were identified as belonging to the cortelezzii complex (*Ev. cortelezzii-sallesi*) because the females are indistinguishable by morphological characteristics.

The geographic coordinates, dates, and results are shown in the Table. *Migonemyia migonei* was reported previously (Salomón et al. 2008), while*Ev. cortelezzii-sallesi* is reported for the first time in the province of Córdoba.

In the Chaco bio-geographical region, the captures and the ACL incidence showed two disparate patterns: (i) time-space scattering of ACL cases (sporadic transmission) in the western dry Chaco, with *Mg. migonei* and *Ev. cortelezzii-sallesi* as the prevalent species of Phlebotominae, and (ii) ACL outbreaks (potential epidemic transmission) in the eastern humid Chaco and gallery forests of rivers associated with *Nyssomyia nei- vai* dominance (Salomón et al. 2001, 2002, 2006b, Rosa et al. 2010, Quintana et al. 2012).

Therefore, the results presented here show the same species pattern in the Espinal region as in the dry Chaco region with sporadic transmission. The Espinal region reaches the Paraná River in the west, where *Mg. migonei* was also reported (31º35’S 60º17’W), while in the residual woods of the dry Chaco far from the river (28º38’S 59º51’W), *N. neivai*, *Ev. cortelezzii*, and *Mg. migonei* were found in very low abundance with no dominance between the species (Salomón et al. 2006a).

Both species found in Córdoba have vectorial competence. *Mg. migonei* is a known vector of *Leishmania braziliensis* (Diniz et al. 2014), already incriminated in Argentina (Quintana et al. 2012, 2013). It was also associated in the Chaco region with the agent of visceral leishmaniasis, *Leishmania infantum*, when the parasite circulation increased and, in the absence of the main vector *Lutzomyia longipalpis* (Salomón et al. 2010), as in the state of Pernambuco, Brazil (de Carvalho et al. 2010), and *L. infantum* DNA was detected in wild-caught specimens from Puerto Iguazú (Moya et al. 2015). Females of the *Ev. cortelezzii* complex were reported with *L. braziliensis* DNA in the Chaco region (Rosa et al. 2012) and *L. infantum* DNA was also found in both species of the complex in the state of Minas Gerais, Brazil (Carvalho et al. 2008, Saraiva et al. 2009).

In the dry Chaco, the best predictor of sandfly abundance is the precipitation (Salomón et al. 2008), and in areas lacking a continuous canopy, even the shadow of land-growing bromeliads are suitable breeding sites (Parras et al. 2012). However, in the humid subtropical areas, the variables that more effectively predicted the distribution*Mg. migonei* were the precipitation of the driest month and the precipitation of the warmest quarter (Quintana et al. 2013). In this sense, it is worth noting that the area of persistent sandfly colonisation (the Mar Chiquita Basin) has cyclical dryness-overflow periods, and the area of new reports close to the city of Córdoba (Unquillo, Córdoba outskirts) has shown changes in the flora and fauna in recent years associated with the reduction of natural habitats (Zak et al. 2004, 2008).

A model showed that the presence of sandflies is better defined by macrohabitat characteristics, while their abundance is associated with microhabitat variables at the site of capture (Santini et al. 2015). Thus, despite the low abundances found, environmental microscale conditions could produce focal outbursts of sandflies, as was found in the Chaco region in Suncho Corral, in the province of Santiago del Estero, where 1,555 *Mg. migonei* and 64*Ev. cortelezzii* were collected in one trap in one night within a horse stable (Salomón et al. 2008).

In the yard of the ACL case diagnosed in Córdoba, a single sandfly was captured, but the ACL patient had been working as a builder during the season of main sandfly activity in Miramar between La Para and Altos de Chipión, a locality that should be re-located after an overflow of the Mar Chiquita Lagoon.

Therefore, these new records close to the known distribution border could be due to (i) intensified awareness and “looking for” sandflies in areas where their abundance is usually low and their presence sporadic, (ii) residual populations from a more extended historical area that is currently fragmented, (ii) sporadic extended colonisation during optimal climate periods but subject to extinctions, or (iv) actual spread through “least-cost” environmental paths (Fischer et al. 2011).

In conclusion, although the low abundance of vectors could imply a low probability of transmission in peridomestic environments, sporadic cases or an emergent trend could occur, or even visceral cases, due to increased parasite circulation. Surveillance in leishmaniasis-vulnerable contiguous areas and receptive areas due to the reported presence of vectors should be intensified, even in temperate climate regions. The spread and colonisation trends of vectors should be monitored, the Health System should be sensitised and enabled to provide early detection and proper treatment of the cases, and each confirmed case will require a study of the focus to assess autochthony.
